# Low Levels of Polymorphisms and No Evidence for Diversifying Selection on the *Plasmodium knowlesi* Apical Membrane Antigen 1 Gene

**DOI:** 10.1371/journal.pone.0124400

**Published:** 2015-04-16

**Authors:** Bart W. Faber, Khamisah Abdul Kadir, Roberto Rodriguez-Garcia, Edmond J Remarque, Frederick A. Saul, Brigitte Vulliez-Le Normand, Graham A. Bentley, Clemens H. M. Kocken, Balbir Singh

**Affiliations:** 1 Department of Parasitology, Biomedical Primate Research Centre, Rijswijk, The Netherlands; 2 Malaria Research Centre, Faculty of Medicine and Health Sciences, Universiti Malaysia Sarawak, Kuching, Sarawak, Malaysia; 3 Institut Pasteur, Unité d’Immunologie Structurale, Département de Biologie Structurale et Chimie, Paris, France; 4 CNRS URA 2185, Paris, France; Université Pierre et Marie Curie, FRANCE

## Abstract

Infection with *Plasmodium knowlesi*, a zoonotic primate malaria, is a growing human health problem in Southeast Asia. *P*. *knowlesi* is being used in malaria vaccine studies, and a number of proteins are being considered as candidate malaria vaccine antigens, including the Apical Membrane Antigen 1 (AMA1). In order to determine genetic diversity of the *ama1* gene and to identify epitopes of AMA1 under strongest immune selection, the *ama1* gene of 52 *P*. *knowlesi* isolates derived from human infections was sequenced. Sequence analysis of isolates from two geographically isolated regions in Sarawak showed that polymorphism in the protein is low compared to that of AMA1 of the major human malaria parasites, *P*. *falciparum* and *P*. *vivax*. Although the number of haplotypes was 27, the frequency of mutations at the majority of the polymorphic positions was low, and only six positions had a variance frequency higher than 10%. Only two positions had more than one alternative amino acid. Interestingly, three of the high-frequency polymorphic sites correspond to invariant sites in PfAMA1 or PvAMA1. Statistically significant differences in the quantity of three of the six high frequency mutations were observed between the two regions. These analyses suggest that the *pkama1* gene is not under balancing selection, as observed for *pfama1* and *pvama1*, and that the PkAMA1 protein is not a primary target for protective humoral immune responses in their reservoir macaque hosts, unlike PfAMA1 and PvAMA1 in humans. The low level of polymorphism justifies the development of a single allele PkAMA1-based vaccine.

## Introduction

In the last decade it has become increasingly clear that the simian malaria parasite, *Plasmodium knowlesi*, is widely distributed in Southeast Asia and can cause severe disease and death in humans [1,2]. Its distribution coincides largely with the distribution of its natural hosts, the long-tailed and pig-tailed macaques, and the Anopheline vectors belonging to the *Anopheles leucosphyrus* group [1]. *P*. *knowlesi* infections in humans were overlooked for a long time as they were misdiagnosed by microscopy as the morphologically similar *P*. *malariae* [2]. Today, *P*. *knowlesi* is considered a serious threat, responsible for approximately 66% of all hospitalized malaria cases for the year 2013 in Malaysian Borneo [3]. Although *P*. *knowlesi* is an ancient parasite that has been infecting humans for a long time [4], such infections are considered to be primarily a zoonotic event. There are no biological barriers that would prevent transmission by mosquitoes from macaques to humans and from humans to humans, and this has been demonstrated under laboratory conditions [5].

The distribution and magnitude of infections by *P*. *knowlesi* warrants the development of a vaccine against *P*. *knowlesi*. Apical Membrane Antigen 1 (AMA1) is a promising vaccine candidate (reviewed in [6]). A recent vaccination trial with rhesus monkeys using heterologously expressed *P*. *knowlesi* AMA1 (PkAMA1) showed that, after two booster immunizations, five out of six animals were able to control the parasitemia [7].

*P*. *falciparum* and *P*. *vivax* AMA1 are highly polymorphic molecules, requiring the development of vaccine strategies to overcome the diversity [8–12]. In order to determine genetic diversity of the *ama1* gene and to identify epitopes on AMA1 under strongest immune selection, the *ama1* gene of 52 *P*. *knowlesi* isolates derived from human infections was sequenced and analysed.

## Methods

Sample collection: Blood samples were collected from *Plasmodium knowlesi* malaria patients at Kapit Hospital and Betong Hospital, Sarawak, Malaysia, after written informed consent was obtained. The distance between Kapit Hospital and Betong Hospital is 233 km. This study was approved by the Medical Ethics Committee of Universiti Malaysia Sarawak. DNA was extracted from the blood samples using the QIAamp DNA Blood Midi Kit (Qiagen, Germany). The *P*. *knowlesi ama1* gene was amplified from 65 samples using primers 2334F, GCTACCTGAACTCGGTTGCTA (starting 102 bp upstream from the start codon ATG) and 2335R, ACACCCACAGTTGTTACGACC (ending 57 bp after the stop codon). PCR amplification was undertaken with the Phusion High-Fidelity DNA Polymerase PCR Kit (Thermo Scientific, USA). Amplification was for 35 rounds, with denaturing at 98°C for 7 s, annealing at 63°C for 20 s and elongation at 72°C for 52 s. PCR products were purified using Gel/PCR DNA Fragment Extraction kit (Geneaid, Taiwan) and eluted using 20 μl of elution buffer.

The same primers were used for sequencing the full-length *pkama1* coding region. Additionally, internal primers 2371F, GGTCCACGATAC and 2370R, TGGTTGATCTGAAGCGCT were used. The latter primers were designed based on preliminary sequencing results which showed that these parts of the gene were fully conserved. Sequencing was performed by Baseclear, Leiden, The Netherlands. Sequences were deposited in the Genbank database [Genbank Accession numbers KP067834-KP067885]. Five samples could not be sequenced with at least one of the four primers used and these were not included in the final analysis. Eight samples showed double signals at positions later identified as polymorphic, indicating multiple infections. These samples were also not included in the analysis.

### Sequence alignments and statistical analysis

DNA and protein sequences were aligned with Macvector 12.7.5 (MacVector Inc., Cary, NC), using the Muscle algorithm [13].

Pi is calculated as the average pairwise nucleotide diversity, while Tajima’s D compares the nucleotide diversity with the total number of mutations [14] and evaluates whether there is statistically significant deviation from neutrality.

Fu and Li's D and F tests: In the D test, (an estimate of) the number of mutations in external branches of the phylogeny is compared with the total number of mutations, while in the F test the external branch mutations are compared with the average pairwise diversity, and deviations from neutrality are evaluated [15].

These analyses were performed on each of the three domains of the ectodomain of *pkama1*, in addition to the whole sequence, which was also analysed using a sliding window approach.

For all the above analyses, DnaSP version 5.10.01 software [16] was used.

## Results

Of the 65 samples that were sequenced, five could not be sequenced with at least one of the four primers used and these were not included in the final analysis. Eight samples showed double signals at positions later identified as polymorphic, indicating multiple infections. These samples were also not included in the analysis. The final data set comprised 52 full-length sequences, 27 from Betong Division (BTG) and 25 from Kapit Division (CDK).

### Analysis on the protein level

**Fig 1:**
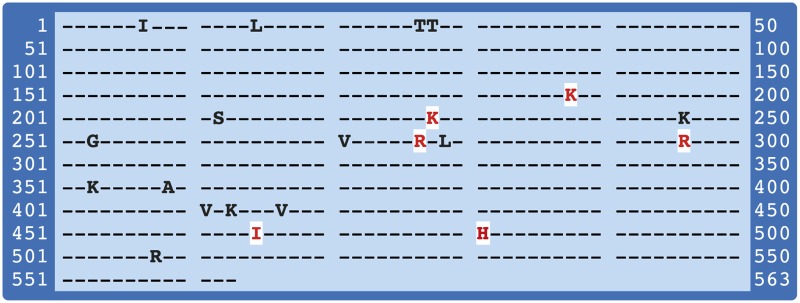
Polymorphic residues in the PkAMA1 protein. High frequency polymorphic sites in red, bold and highlighted in white; low frequency polymorphic sites in black.

The translated DNA sequences were aligned (Fig 1) and 27 haplotypes were identified from the protein sequences (S1 Table). One haplotype occurred nine times, the next most frequent haplotype occurred six times, then two occurring five times, one occurring four times, one occurring three times, four occurring twice and finally 17 occurred only once (S1 Table). Interestingly, 11 haplotypes were found exclusively in Kapit Division, another 11 exclusively in Betong Division.

Twenty-one positions were identified as polymorphic, most with low frequency. Two of these were found in the relatively short signal sequence (aa1-aa24) and two in the even shorter prodomain (aa25-aa42) of PkAMA1. Four polymorphic positions were found in domain I (aa43-aa248), seven in domain II (aa249-aa385), and five in domain III (aa386-aa487) [17]. Finally, one mutation was found in the cytoplasmic tail (Table 1).

**Table 1:** Polymorphism in the PkAMA1 protein, total and per district.

aa Position	Total (52)			Betong (27)			Kapit (25)			Domain
	aa1	aa2	aa3	aa1	aa2	aa3	aa1	aa2	aa3	
7	I (51)	V (1)		I (26)	V (1)		I (25)	-		Signal
15	L (50)	I (1)	V (1)	L (26)	I (1)	-	L (24)	V (1)	-	Signal
27	T (47)	N (5)		T (24)	N (3)		T (23)	N (2)		Pro
28	T (50)	N (2)		T (26)	N (1)		T (24)	N (1)		Pro
**188**	**K (45)**	**N (7)**		K (26)*	N (1)*		K (19)*	N (6)*		**I**
212	S (51)	L (1)		S (27)	-		S (24)	L (1)		**I**
**228**	**K (31)**	**N (21)**		**K (16)**	**N (11)**		**K (15)**	**N (10)**		**I**
246	K (51)	R (1)		K (26)	R (1)		K (25)	-		**I**
253	G (51)	R (1)		G (26)	R (1)		G (25)	-		**II**
271 ^#^	V (50)	F (2)		V (26)	F (1)		V (24)	F (1)		**II**
**277**	**R (45)**	**T (7)**		R (26)*	T (1)*		R (19)*	T (6)*		**II**
279	L (51)	V (1)		L (26)	V (1)		L (25)	-		**II**
**296**	**R (35)**	**S (17)**		**R (21)**	**S (6)**		**R (14)**	**S (11)**		**II**
353	K (50)	R (2)		K (26)	R (1)		K (24)	R (1)		**II**
359	A (51)	T (1)		A (27)	-		A (24)	T (1)		**II**
411	V (49)	A (3)		V (25)	A (2)		V (24)	A (1)		**III**
413	K (51)	R (1)		K (26)	R (1)		K (25)	-		**III**
417	V (51)	I (1)		V (26)	I (1)		V (25)	-		**III**
**465**	**I (38)**	**F (14)**		I (23)*	F (4)*		I (15)*	F (10)*		**III**
**481**	**H (22)**	**N (19)**	**Q (11)**	**H (11)**	**N (12)**	**Q (4)**	**Q (10)**	**H (8)**	**N (7)**	**III**
508	R (51)	T (1)		R (27)	-		R (24)	T (1)		**CT**

In parentheses the number of amino acids (aa) found. High frequency polymorphic sites are in bold. Sites corresponding with polymorphic sites in PvAMA1 or PfAMA1 are underlined.

*statistically significant difference

^#^present in PfAMA1 at very low frequency (0.1%)(2 in 1920 sequences, EJR, personal communication)

Only seven of these polymorphic positions correspond to polymorphic sites identified in PfAMA1, two of which were also found in PvAMA1 (aa228 and aa277) [17,18]. Three of these seven sites were found in domain I, the remaining four in domain II. One of the corresponding sites in PfAMA1 has a very low frequency (0.1%) (Table 1).

**Fig 2:**
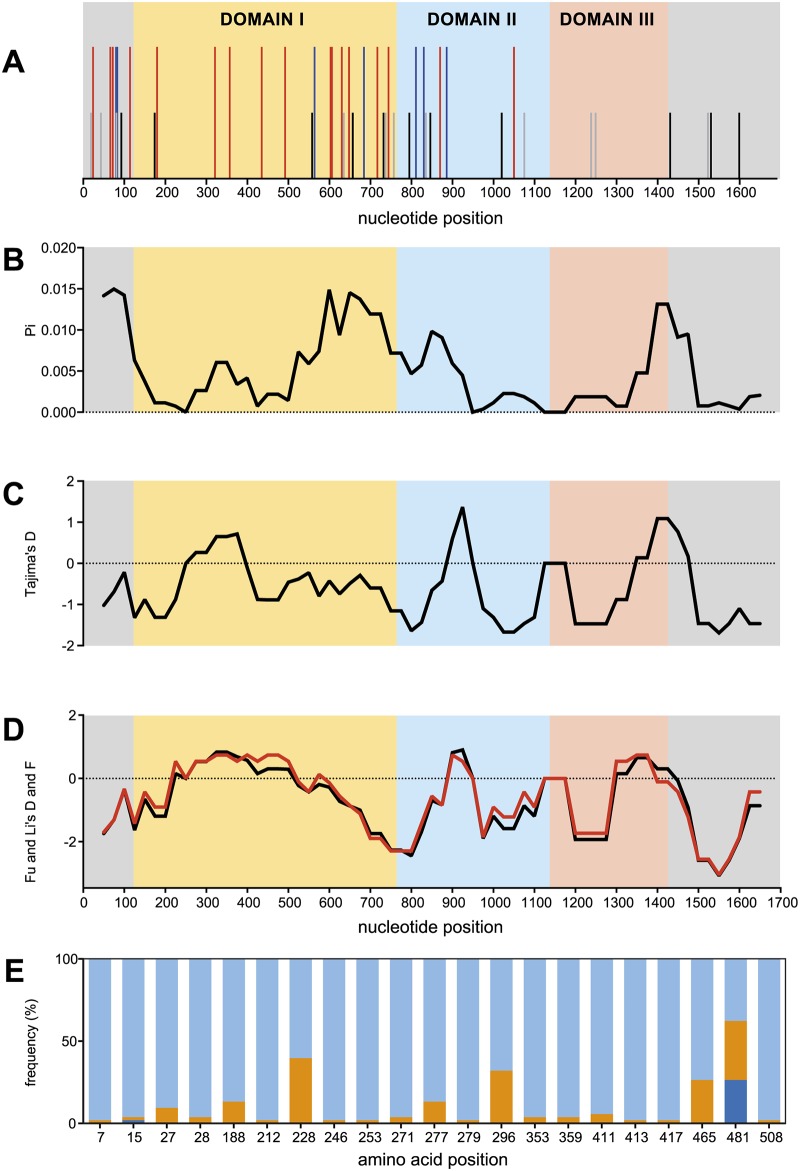
Analyses of the genes encoding the full length PkAMA1 protein. A) Schematic representation of the *pkama1* gene, indicating the locations of all non-synonymous (NS, red lines), synonymous (SP, blue lines) and NS and SP singleton (black and grey lines, respectively) SNPs. B) Sliding window analysis showing the average pairwise nucleotide diversity (Pi) values in Pkama1 for the 52 sequences included in the analysis. A window size of 100 bp and a step size of 25 bp were used. C) Sliding window calculation of Tajima’s D. A window size of 100 and a step size of 25 were used. D) Sliding window calculation of Fu&Li’s D (red line) and F (blue line). A window size of 100 and a step size of 25 were used. E) Amino acid frequencies. The frequencies of 21 amino acid polymorphic positions are indicated by the proportion of each bar and its color. Light blue, most prominent amino acid; orange, second most prominent amino acid; dark blue, least prominent amino acid.

Six high frequency polymorphic sites (f >10%) were identified (Table 1, Fig 2E). Three of these are shared with PfAMA1 at the following positions: Pkaa188(KN)→Pfaa243(KNE), Pkaa228(KN)→Pfaa283(SL) and Pkaa277(RT)→Pfaa332(NI), the latter two also with PvAMA1 [17,18]. The three remaining high frequency polymorphisms occur neither in PfAMA1 nor in PvAMA1. Of these, residue aa481 and aa465 are very close to the membrane-spanning region. On basis of homology with PfAMA1, these sites are located outside the normal cleavage site of the shedding protease PfSUB1 (PfAMA1 T517, corresponding to K459 in PkAMA1 [19]). The third high-frequency polymorphic site in PkAMA1 that does not occur in PfAMA1 is R296. This residue is at a stem region of the flexible loop in domain II, and contributes a salt bridge. The implications of the PkAMA1 mutations for structure/function are discussed elsewhere [20].

Interestingly, no polymorphic residues are found in the flexible loop in domain II (bp 891–1058, corresponding to aa297-352). This sequence comprises the highly conserved loop in PfAMA1 domain II and is involved in the binding process of the RON2 protein during invasion of the red blood cell [21]. Other longer invariant regions in the PkAMA1 gene (bp 181–320; bp 1076–1231 and bp 1250–1349) do not have conserved counterparts in Pf- or PvAMA1.

**Fig 3:**
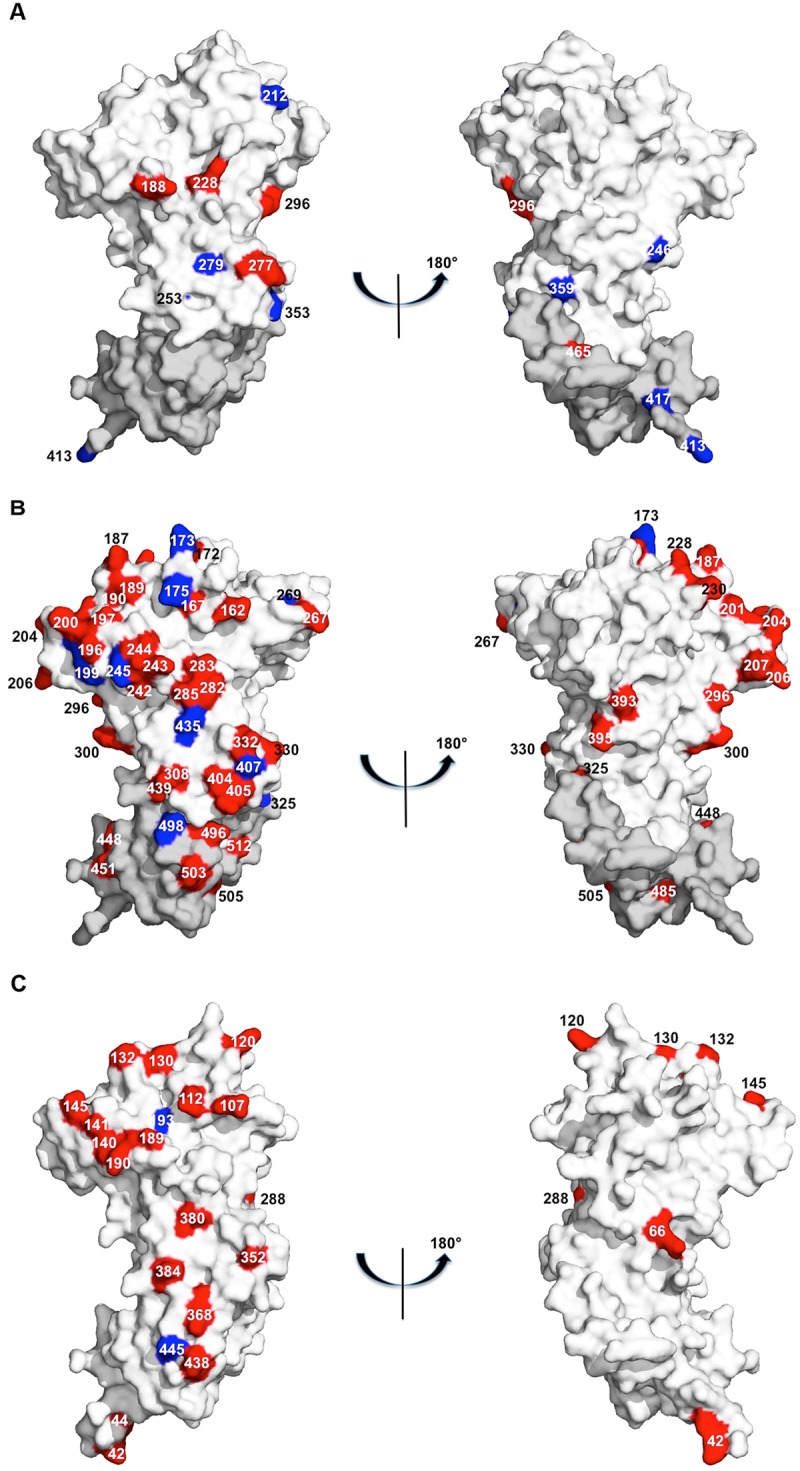
Three-dimensional distribution of polymorphic amino acid residues of PkAMA1, PfAMA1 and PvAMA1. Two views of a surface representation, rotated by 180° with respect to each other, are shown for the crystal structures of (A) PkAMA1 (PDB entry 4UV6), (B) PfAMA1 (PDB entry 2Z8V) and (C) PvAMA1 (PDB entry 1W8L), with each orthologue oriented at equivalent angles. PkAMA1 polymorphisms are from this study, PfAMA1 polymorphisms are from refs [23,24] and PvAMA1 polymorphisms are from ref [25]. Polymorphic residues are labeled and colored in red for high frequency polymorphisms (> 10%) and in blue for low frequency polymorphisms (< 10%). Domains 1 and 2 from crystal structures of all three orthologues are shown in white. Domain 3, shown in grey, was modeled for PkAMA1 and PfAMA1 from the crystal structure of PvAMA1 since the crystal structure of this domain has not been determined for these two orthologues. (PkAMA1 Domain 3 polymorphic residues 411 and 481 are not shown as these are disordered and thus not visible in the PvAMA1 structure).

Fig 3 shows the high and low frequency polymorphic amino acid residues on the PkAMA1 crystal structure [20] and compares them with those of PvAMA1 and PfAMA1. From these three-dimensional views, the differences between the proteins are clear: both PvAMA1 and PfAMA1 have more high-frequency polymorphic residues on the ectodomain of the protein, and while PvAMA1 has more polymorphic positions on the ectodomain compared to PkAMA1, PfAMA1 polymorphisms outnumber both of them by a significantly large margin. Note that the high-frequency residues preferentially locate to one face of the protein [22].

Comparing the frequency of the alternative amino acid residues between isolates derived from Kapit and Betong Divisions (Table 1) reveals a statistically significant difference for three high frequency positions [Fisher’s exact, P< 0.05].

### Analysis on the DNA level

From the alignment of the 52 *pkama1* genes, 47 different allele sequences were identified. As visualized in Fig 2A, a high number of singleton substitutions were found, either synonymous or non-synonymous.

Pi-values, Tajima’s D and Fu & Li’s D and F values were determined for the full-length sequences (Table 2). The Pi-value, the average pairwise nucleotide diversity per site, was 0.00501 for the whole gene. This is low in comparison to *pfama1* [23,24] and indicates that the sequences do not differ to a large extent. The other parameters (Tajima’s D and Fu & Li’s D and F) are indicators for deviations from the neutrality hypothesis, i.e. they are used to assess whether or not there is selective pressure on the gene. Although the values of these indicators are all below -1.0, they do not differ statistically significantly from zero.

**Table 2:** Summary of sequence analysis for 52 PkAMA1 genes and tests for departure from neutrality.

	S	S (Si)	H	Pi	Tajima’s D	Fu&Li’s D	Fu&Li’s F
**Total region**	55 (58)	23	46	0.00501	-1,17581	-1,84397	-1,90697
**Domain 1**	19 (21)	6	36	0,00586	-0,97724	-0,81587	-0,92129
**Domain 2**	12 (12)	6	11	0,00313	-1,51276	-1,79651	-2,01006
**Domain 3**	8 (8)	3	9	0,00515	-0,29598	-0,85941	-0,79627

The full-length sequence of each gene was obtained. Domain 1: aa43-248 = bp127-744; Domain 2: aa249-385 = bp745-1155; Domain 3: aa386-487 = bp1156-1461 [17]. S, number of segregating sites (in parentheses the number of mutations); S (Si), number of singleton mutations; H, number of haplotypes; Pi, average pairwise nucleotide diversity.

As selective pressure may act on specific regions of the protein, a sliding window approach for the above parameters was taken (Fig 2B–2D). This is of particular interest for the *ama1* gene as the protein consists of three distinct domains and *pfama1* domain II has been shown not to be under balancing selection [24]. Fig 2 shows that none of the domains in *pkama1* exhibit significant deviations from the null-hypothesis (see also Table 2), although all the calculated parameters were below zero.

## Discussion

Analysis of the translations of the 52 *pkama1* gene sequences showed that the overall amino acid polymorphism of PkAMA1 is low compared to that of PfAMA1. Twenty-one amino acid positions were found to be polymorphic, against 41 (n = 50) and 53 (n = 51) in two comparable studies of PfAMA1 [23,24]. The frequency of the occurrence of alternative amino acids in the PkAMA1 sequences was also low, as this frequency was higher than 10% at six amino acid positions only, while single mutations were found at 10 amino acids positions. In PfAMA1 the number of amino acid positions with polymorphic frequencies higher than 10% was 39 (of 41) in Thai subjects and 43 (of 53) positions in Nigerian subjects [23,24].

A similar study on 73 *pvama1* sequences gathered in the Venezuelan Amazon revealed 22 amino acid polymorphic sites, of which 20 showed a frequency greater than 10% [25]. The total number of polymorphisms in PvAMA1 is thus similar to those found in PkAMA1. However, the frequencies of the alternative amino acids are much higher for PvAMA1 compared to PkAMA1. The distribution of polymorphic amino acid sites in the three-dimensional structures of PvAMA1, PfAMA1 and PvAMA1 are compared in Fig 3 and illustrates the above description.

The low polymorphism observed in PkAMA1 suggests that a single allele could serve as a blood stage vaccine. In PfAMA1 the use of a single allele is problematic and mixtures of three or more alleles are currently being pursued to overcome the high levels of polymorphism [8,11,26,27].

Analysis at the DNA level shows that the average pairwise nucleotide diversity (Pi) between the PkAMA1 sequences is low, overall as well as per domain. The values are also much lower in comparison to those observed between PfAMA1 sequences. Although not statistically significantly different from the neutral hypothesis, the calculated parameters Tajima’s D, Fu & Li’s D and F have strong negative values for the full gene. Strong negative signs for the Tajima’s D/Fu and Li’s D and F point towards either a purifying selection or a population expansion [23,24]. Two similar-sized PfAMA1 sequence analysis studies show strongly positive Tajima’s D and Fu and Li’s F values. From these studies, it was inferred that balancing selection acts on the *pfama1* (and *pvama1*) gene, in particular on domain I and domain III [23–25].

The negative sign of these parameters in *pkama1* is the result of a high number of singleton substitutions present in the gene (Fig 2A) that result in a small Pi-value, while the total diversity (Θ) is much higher. This pattern is quite distinct from that observed in PvAMA1 [17,18,25] and PfAMA1 [17,23,24].

On basis of the calculated (negative) values of the parameters (Tajima’s D, Fu&Li’s D & F), it is clear that the balancing selection observed for *pfama1* and *pvama1* (with strong positive D and F-values) is not present in the *pkama1* gene. However, care must be taken in the interpretation of these kinds of analyses, as they are based on a number of assumptions [14,15]. For both Tajima’s D and Fu&Li’s D and F, the calculated parameters can essentially be seen as the sum of all individual sites. The six amino acid positions with higher frequencies of variation may therefore be under balancing selection, while the other, low frequency polymorphic positions may be under purifying selection, in total resulting in a negative value pointing to purifying selection. An example for the limitations of the statistical analysis is the antigen escape region in domain II of PfAMA1 [28], a domain that, as a whole, has been described as not being under balancing selection [23–25]. Purifying selection has been observed before for other malarial antigens [29,30].

The *P*. *knowlesi* isolates that were used for DNA sequencing were derived from human patients. As *P*. *knowlesi* malaria is considered to be primarily a zoonosis, they originate from macaques. Therefore, assuming that the sequenced *pkama1* genes are fully representative of the *pkama1* gene pool present in macaques (i.e. not just a select subset that can infect humans), the sequence diversity we report here results from the interaction between the parasite and its monkey host. If so, the difference between the observed purifying selection in *pkama1* and balancing selection in *pfama1* and *pvama1* reflects intrinsic differences in the immunological targets that are used by the respective host species. In this respect it is interesting to note that the PkAMA1 protein was successfully used as a vaccine in rhesus macaques (*M*. *mulatta*) [7].

Sequence analysis of *pkama1* gene isolates and determination of anti-PkAMA1 antibody levels in serum from macaques from the same areas is clearly warranted to understand the factors driving its polymorphism. From the Genbank database only three PkAMA1 sequences are known. The origin of the H strain is human [31] while that of the Nuri strain is a kra monkey (*M*. *fascicularis*), both strains coming from Peninsular Malaysia [32]. We could not find the origin of the W (Washington) strain.

We have sequenced the *pkama1* gene from three other non-human *P*. *knowlesi* isolates, originally obtained from Prof. W. Collins, CDC, Atlanta. Two were isolates from macaques, one from Malaysia and one from the Philippines. The third was isolated from an *A*. *hackeri* mosquito from Peninsular Malaysia [33]. Sequence alignment shows that there are only three amino acid differences among the four non-human isolates. One is at position 228 and two are in the cytoplasmic tail (aa528 and aa555); the latter two mutations are not present in the 52 genes sequenced in this study. The high degree of conservation in the ectodomain is remarkable, especially because these isolates were obtained from different hosts, different locations and different time points. If such a degree of conservation among non-human isolates is confirmed with a larger number of sequences, it will be difficult to explain the variation observed in human hosts without assuming human-to-human transmission. Of note, the same high degree of conservation is observed in the *pkama1* genes from W1 and H strains; these compare very well to the four sequences derived from non-human isolates. This observation points to the possibility that time may also be a significant factor, as the W, H and Nuri strains (as well as the non-human isolates) were isolated halfway through the last century. If the gene diversified from this time onwards, this coincides with the increased human presence in formerly forested areas [34]. Interestingly, Putaporntip *et al*. have shown that, in a limited study, PkMSP1 sequences from human patients in Thailand are more diverse than those obtained from local monkeys [35], and they suggest that this could be due to human-to-human infections.

An alternative explanation for a negative Tajima’s D (and F&L’s D and F) would be to assume an expanding population. Analysis of the mitochondrial DNA sequences of *P*. *knowlesi* derived from macaques and humans in the Kapit Division indicated that *P*. *knowlesi* underwent a population expansion 30,000 to 40,000 years ago of which the exact nature remains elusive [34]. The obvious candidate for a contemporary population expansion would be into the human population, suggesting human-to-human infection. More research is clearly needed to investigate this.

Statistically significant differences were observed between the frequencies of three of the six high frequency polymorphic sites in samples derived from Kapit versus Betong Division. The reason for these differences will remain elusive until it has been determined whether or not these sites are under balancing selection. If they are, the obvious reason would be that the systems are not in equilibrium, i.e. the monkey populations are not in close contact with each other as the distance between Kapit and Betong Hospital is 233 km, and mosquitoes do not travel long distances [36]. For *P*. *falciparum* the amino acid frequencies at polymorphic positions also differ between different geographical areas [23,24].

In summary, the sequence data point to the absence of conclusive evidence for wide-spread balancing selection for the *pkama1* gene from human isolates that was observed for *pfama1* and *pvama1*. Further research will reveal whether *pkama1* genes derived from parasites in the macaque hosts have the same characteristics, while sequencing a limited set of other selected genes (under balancing selection or not) from both macaque- and human-derived samples may reveal more interesting aspects of gene diversity in *P*. *knowlesi* and also may give additional clues whether man-to-man transmission has been established in the field. The low polymorphism in the PkAMA1 protein warrants vaccine development for *P*. *knowlesi* with a single PkAMA1 allele.

## Supporting Information

S1 Table Haplotypes identified in each of the divisions. BTG = Betong, CDK = Kapit Division.
(DOCX)
