# Supplementary material for: Low Levels of Polymorphisms and No Evidence for Diversifying Selection on the Plasmodium knowlesi Apical Membrane Antigen 1 Gene
Source: PLoS One. 2015 Apr 16;10(4):e0124400. doi: 10.1371/journal.pone.0124400 (PMC4400157; doi:10.1371/journal.pone.0124400)
Supplement: S1 Table — BTG = Betong, CDK = Kapit Division. (DOCX) [file pone.0124400.s001.docx]

**Table S1. Haplotypes identified in each of the divisions. BTG = Betong, CDK = Kapit Division.**

|  | **Number of Occurences** | | |  |
| --- | --- | --- | --- | --- |
| **Haplotype #** | **BTG** | **CDK** | **Total** | **% of Total** |
| **1** | 5 | 4 | 9 | 0,173 |
| **2** | 5 | 1 | 6 | 0,115 |
| **3** | 2 | 3 | 5 | 0,096 |
| **4** | 3 | 1 | 4 | 0,077 |
| **5** | 1 | 2 | 3 | 0,058 |
| **6** | 0 | 2 | 2 | 0,038 |
| **7** | 0 | 2 | 2 | 0,038 |
| **8** | 0 | 2 | 2 | 0,038 |
| **9** | 0 | 1 | 1 | 0,019 |
| **10** | 0 | 1 | 1 | 0,019 |
| **11** | 0 | 1 | 1 | 0,019 |
| **12** | 0 | 1 | 1 | 0,019 |
| **13** | 0 | 1 | 1 | 0,019 |
| **14** | 0 | 1 | 1 | 0,019 |
| **15** | 0 | 1 | 1 | 0,019 |
| **16** | 0 | 1 | 1 | 0,019 |
| **17** | 1 | 0 | 1 | 0,019 |
| **18** | 1 | 0 | 1 | 0,019 |
| **19** | 1 | 0 | 1 | 0,019 |
| **20** | 1 | 0 | 1 | 0,019 |
| **21** | 1 | 0 | 1 | 0,019 |
| **22** | 1 | 0 | 1 | 0,019 |
| **23** | 1 | 0 | 1 | 0,019 |
| **24** | 1 | 0 | 1 | 0,019 |
| **25** | 1 | 0 | 1 | 0,019 |
| **26** | 1 | 0 | 1 | 0,019 |
| **27** | 1 | 0 | 1 | 0,019 |
